# The International Myopia Institute (IMI) and consensus building

**DOI:** 10.1111/opo.13458

**Published:** 2025-02-17

**Authors:** Nina Tahhan, Christine Wildsoet, Weizhong Lan, Serge Resnikoff

**Affiliations:** ^1^ Brien Holden Vision Institute (BHVI) University of New South Wales Sydney Australia; ^2^ School of Optometry and Vision Science, Faculty of Medicine University of New South Wales Sydney Australia; ^3^ Herbert Wertheim School of Optometry & Vision Science University of California Berkeley California USA; ^4^ Aier Academy of Ophthalmology Central South University Changsha Hunan China

In recent decades, there has been remarkable progress in myopia research and management, with significant advances in understanding its underlying causes and strategies to modify its onset and progression. However, as with any evolving discipline, the rapid pace of discovery and innovation often gives rise to diverse and sometimes conflicting viewpoints. This special issue of OPO provides a platform for exploring these divergent viewpoints through a series of point‐counterpoint articles on key topics in myopia. These topics were debated by the contributing authors at the 2025 International Myopia Conference (IMC) and coordinated by the International Myopia Institute (IMI).

The IMI was founded after a joint meeting between the World Health Organization (WHO) and the Brien Holden Vision Institute (BHVI) in Sydney, Australia, in 2015. This occurred at a time when the results of the first large global meta‐analysis on the prevalence of myopia, eventually published in 2016,^1^ made it apparent that myopia prevalence was growing at an alarming rate around the globe and more needed to be done to address the growing global impact. A total of 23 scientific and clinic experts in myopia from all six WHO regions came together, and consensus was reached on a series of definitions, statements and recommendations. It was agreed that addressing significant gaps in knowledge was urgently needed to establish evidence‐based strategies for reducing high myopia and associated vision impairment. In the interest of progress, the need to foster collaborations among researchers, clinicians and policymakers was also recognised, paving the way for the formation of the IMI.

Since its inception, the IMI has played a pivotal role in fostering the collaborations mentioned above and synthesising evidence‐based consensus on myopia‐related topics through initiatives such as its seminal white paper series and biennial digests. These documents, authored by leading experts from across the myopia landscape, have served as important resources for guiding research, clinical best practice and public health intervention strategies. Despite the significant progress and many achievements in this field, certain topics, including ones directly related to myopia management, remain contentious. This largely reflects the complexity of the underlying science and its practical application. Recognising this, the IMI identified several key topics for debate at the recent IMC, aiming to showcase opposing yet evidence‐supported viewpoints and foster further dialogue where consensus is still needed.

This collection of point‐counterpoint articles captures the spirited discussions from the IMC while offering readers a comprehensive overview of the merits and challenges associated with each debate topic. Each article includes independently written, concise summaries that distil the key arguments, highlight points of agreement and identify unresolved issues, serving as potential roadmaps for future research.

In summary, we hope you enjoy reading these articles as much as the audience enjoyed the live debates. We also hope this special issue serves as both a reflection of the vibrant myopia research and clinical communities and a catalyst for advancing our shared goal: developing effective, equitable and evidence‐based strategies to manage the myopia epidemic.
